# Insights into the evolution of pathogenicity of *Escherichia coli* from genomic analysis of intestinal *E. coli* of *Marmota himalayana* in Qinghai–Tibet plateau of China

**DOI:** 10.1038/emi.2016.122

**Published:** 2016-12-07

**Authors:** Shan Lu, Dong Jin, Shusheng Wu, Jing Yang, Ruiting Lan, Xiangning Bai, Sha Liu, Qiong Meng, Xuejiao Yuan, Juan Zhou, Ji Pu, Qiang Chen, Hang Dai, Yuanyuan Hu, Yanwen Xiong, Changyun Ye, Jianguo Xu

**Affiliations:** 1 State Key Laboratory for Infectious Disease Prevention and Control, and National Institute for Communicable Disease Control and Prevention, Chinese Center for Disease Control and Prevention, Beijing 102206, China; 2 Collaborative Innovation Center for Diagnosis and Treatment of Infectious Diseases, Hangzhou 310003, Zhejiang Province, China; 3 Yushu Prefecture Center for Disease Control and Prevention, Qinghai province, Yushu 815000, Qinghai Province, China; 4 School of Biotechnology and Biomolecular Sciences, University of New South Wales, Sydney 2052, New South Wales, Australia

**Keywords:** *Escherichia coli*, evolution, Marmot, pathogenicity, virulence genes

## Abstract

*Escherichia coli* is both of a widespread harmless gut commensal and a versatile pathogen of humans. Domestic animals are a well-known reservoir for pathogenic *E. coli*. However, studies of *E. coli* populations from wild animals that have been separated from human activities had been very limited. Here we obtained 580 isolates from intestinal contents of 116 wild Marmot *Marmota himalayana* from Qinghai–Tibet plateau, China, with five isolates per animal. We selected 125 (hereinafter referred to as strains) from the 580 isolates for genome sequencing, based on unique pulse field gel electrophoresis patterns and at least one isolate per animal. Whole genome sequence analysis revealed that all 125 strains carried at least one and the majority (79.2%) carried multiple virulence genes based on the analysis of 22 selected virulence genes. In particular, the majority of the strains carried virulence genes from different pathovars as potential 'hybrid pathogens'. The alleles of eight virulence genes from the Marmot *E. coli* were found to have diverged earlier than all known alleles from human and other animal *E. coli*. Phylogenetic analysis of the 125 Marmot *E. coli* genomes and 355 genomes selected from 1622 human and other *E. coli* strains identified two new phylogroups, G and H, both of which diverged earlier than the other phylogroups. Eight of the 12 well-known pathogenic *E. coli* lineages were found to share a most recent common ancestor with one or more Marmot *E. coli* strains. Our results suggested that the intestinal *E. coli* of the Marmots contained a diverse virulence gene pool and is potentially pathogenic to humans. These findings provided a new understanding of the evolutionary origin of pathogenic *E. coli*.

## INTRODUCTION

*Escherichia coli* is both of a widespread harmless gut commensal and a versatile pathogen of humans, which is estimated to cause more than two million deaths annually through both intestinal and extraintestinal infections.^1^ On the basis of the mode of pathogenesis and presence of typical virulence factors, there are at least five well recognized pathovars of intestinal pathogenic *E. coli*, including enteropathogenic, enterohaemorrhagic, enteotoxigenic, enteroaggregative and enteroinvasive *E. coli* (EPEC, EHEC, ETEC, EAEC and EIEC, respectively) and two well recognized pathovars of extraintestinal pathogenic *E. coli* (uropathogenic (UPEC) and Neonatal meningitis *E. coli* (NMEC)).^1^ The major virulence factors for these pathogens are all on mobile elements. EPEC strains carry the LEE pathogenicity island encoding a type III secretion system for effacing and attachment, and the EAF plasmid for adherence. EHEC strains also carry LEE but with two crucial additional elements, Shiga toxin phage(s) and an EHEC plasmid encoding a haemolysin. Shiga toxin *E. coli* (STEC) which may or may not carry LEE is recognized as an extension of EHEC with variable clinical outcomes. ETEC strains contain plasmid borne genes for enterotoxins and specific pili for adhesion. EIEC and *Shigella* strains carry the pINV invasion plasmid and are one pathogenic form as they share the same mode of cell invasion. The typical strains of a pathovar can be defined by one or a few virulence genes.^2, 3^ However, the pathogenicity of UPEC and NMEC is more difficult to define using virulence genes as many genes are implicated.^4, 5^ It seems that UPEC pathogenicity is determined by the total virulence gene content and certain combinations of multiple virulence genes.^4, 5^

However, there are emerging hybrid forms of pathogenic *E. coli* which carry novel combinations of known virulence factors as highlighted by the Shiga toxin producing EAEC O104:H4 which caused the German hemolytic uremic syndrome outbreak.^6^ Other novel EHEC/EAEC hybrid strains and STEC/ETEC hybrid strains have been reported recently.^7, 8^ There are also UPEC strains carrying intestinal pathogenic virulence factors including EAEC and STEC virulence genes.^9^

The established view is that pathogenic *E. coli* was evolved from commensal *E. coli* in humans or animals through horizontal transfer of virulence genes, resulted into various pathovars to cause clinically and epidemiologically distinctive diseases.^10^ Domestic and other animals are natural reservoirs for some human pathogenic *E. coli* with the typical example of STEC, which normally does not cause disease in these animals. On the other hand, the human ETEC and domestic animal ETEC strains are differentiated by their adhesion factors and as a result, human ETEC strains normally do not cause disease in domestic animals and vice versa.^11^ The major human ETEC lineages are estimated to have arisen less than 200 years ago through acquisition of plasmid encoded virulence genes.^12^

In this study, we found that the majority of the 125 strains of intestinal *E. coli* from 116 healthy *M. himalayana* in Qinghai–Tibet plateau, China, carried 22 virulence genes examined including LEE-encoded intimin gene (*eaeA*), the K88 fimbriae usher gene (*faeD*), the EAEC heat-stable toxin 1 gene (*astA*), the EHEC hemolysin gene (*ehxA*), the Shiga toxin 1 gene (*stx1*), the IrgA homolog adhesin gene (*iha*), the long polar fimbria genes (*lpfA*_O113_, and *lpfA*_O157/OI-141_), the 60-MDa virulence plasmid adhesion gene (*toxB*), P fimbriae usher gene (*papC*), the brain microvascular endothelial cell invasion gene (*ibeA*), the cytolethal distending toxin B gene (*cdtB*) and the *Shigella* pathogenicity island encoded gene (*shiA*).^2^ Phylogenetic analysis indicated that for eight virulence genes such as *eaeA*, the alleles found in Marmot *E. coli* diverged earlier than all known alleles from human and other animal *E. coli*.

## MATERIALS AND METHODS

### Marmot sampling, virulence gene screening and strain isolation

The Marmots (*M. himalayana)* were sampled as part of the animal plague surveillance program conducted in Yushu Tibetan autonomous prefecture, Qinghai province. Of a total of 116 Marmots sampled in June 2012, 38 were from Haxiu county (with an altitude of 4116.5 m above sea level (a.s.l)) and 78 from Anchong site (4525.3 m a.s.l). The human population density in Yushu area in 2013 was 1.43 person/km^2^. The distance from sampling sites, Haxiu and Yushu, to the nearest village is 10 km and 15 km, respectively. The Marmots were captured by cages in the field and sampled in the laboratory of local Centre for Disease Control (CDC). The sampling was performed in accordance with the protocol for national plague surveillance program in animals. The study has been reviewed and approved by the ethic committee of National Institute for Communicable Diseases Control and Prevention, China CDC.

The intestinal contents were collected in 2 ml sterile tubes containing Luria-Bertani (LB) medium in 30% glycerol, which were stored at −20 °C immediately and transported to the laboratory in Beijing. The intestinal contents were screened for 22 virulence genes of pathogenic *E. coli*, including EPEC LEE pathogenicity island-encoded intimin *eaeA* gene, *stx1*, *ehxA*, *efa1*, *iha*, *lpfA*_O113_, and *lpfA*_O157/OI-141_, *toxB*, *paa*, *faeD*, *espP*, *etpD*, *astA*, *pic*, *papC*, *tsh*, *hlyA*, *aslA*, *ibeA*, the outmembrane protein Hek gene (*hek*), the polysialic acid capsule transport protein gene (*kpsD*), *cdtB* and *shiA.*^13^

### Whole-genome sequencing and sequence analysis

The genomes were obtained by Illumina sequencing by constructing two paired-end (PE) libraries with average insertion length of 500 and 2000 bp. Reads were generated with Illumina Solexa GA IIx (Illumina, San Diego, CA, USA) and assembled into contigs and scaffolds using SOAP *de novo* with an average depth of coverage of 100 ×. The core genes from 179 genomes including 125 Marmot *E. coli* genomes and 54 *E. coli* genomes from GenBank were extracted, concatenated and aligned using musle.^14^ Whole genome neighbor-joining tree was inferred using Phylip.^15^ The resulting phylogeny was edited using Figtree (http://tree.bio.ed.ac.uk/software/figtree), iTOL^16^ and Adobe illustrator (San Jose, CA, USA).

### Virulence gene sequence phylogenetic analysis

The virulence gene phylogeny was constructed by aligning the nucleotide sequences of each allele using ClustalW alignment algorithm in MEGA5.2.^17^ Neighbor-joining trees were constructed in MEGA5.2 using the Kimura 2-parameter substitution model with 1000 bootstrap replications.

### Population structure estimation

We used population structure analysis software STRUCTURE to identify different *E. coli* groups and subgroups.^18^ We ran 20 000 iterations, following a burn-in period of 10 000 iterations with *K* (number of groups) between 2 and 11 and chose the most suitable *K* when the model probability increased dramatically. A cutoff value of *q*⩾0.67 (*q*: the combined probability of ancestry from each of groups for each of individual isolate) was used to assign individual isolates to their corresponding groups or subgroups.

## RESULTS

### Virulence gene content of intestinal *E. coli* from *M. himalayana*

A total of 116 healthy *M. himalayana* were captured and sampled from Qinghai–Tibet plateau of China in 2012. By using simplex PCR to directly examine the intestinal contents of animals for *E. coli* virulence genes, we found that all 116 *M. himalayana* were positive for at least one, and the majority were positive for multiple of the 22 virulence genes, suggesting that all Marmots carried pathogenic *E. coli* (Table 1).

To test this hypothesis, we obtained five *E. coli* colonies per animal to get a good representation of the intestinal *E. coli* population for a total of 580 isolates. Pulse field gel electrophoresis (PFGE) analysis of chromosomal fragments generated by restriction enzyme *Xba*I digestion showed that the majority of the isolates from the same animal were identical (data not shown). Therefore, one isolate for each PFGE type and at least one isolate for each Marmot were selected for a final set of 125 to represent the 580 isolates. These isolates (hereinafter referred to as strains) were then sequenced using Illumina paired-end 100 bp sequencing and assembled using SOAP *de novo* (GeneBank JZMB00000000-JZQV00000000).

**Figure 1:**
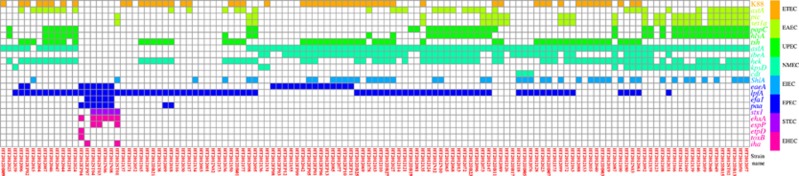
Heat map of 22 virulence genes in 125 intestinal *E. coli* strains of *M. himalayana.* The presence of specific virulence gene was indicated by filled square with pathovar-specific colors as shown in the color legend. The strain numbers were listed at the bottom of panel.

Genomic analysis revealed that the prevalence of the 22 virulence genes examined in Marmot *E. coli* strains varied from 0.80% to 76.80% with an average of 24.80%, while the number of virulence genes carried per strain varied from one to nine genes with an average of 5.46 (Figure 1). Unexpectedly, 41.68% of the Marmot *E. coli* strains contained the porcine ETEC K88 fimbriae gene *faeD*. Nearly all strains contained one or more of the NMEC and/or UPEC genes. The EIEC/*Shigella* invasion plasmid encoded virulence gene *ipaH* was not detected. *ShiA* which is only present in a proportion of *S. flexneri* strains was found in a subset of the Marmot *E. coli* strains. Our results showed that the majority of Marmot *E. coli* strains (92.8%) contained multiple virulence genes.

Pathogenic *E. coli* strains can be classified into pathovars by the presence of specific virulence genes. The classification of EPEC, EHEC and STEC is relatively straightforward while for UPEC and NMEC it is less clear-cut as many genes have been reported to be promiscuously associated with these two pathovars.^19^ We used the presence of the following 15 representative virulence genes detected in Marmot *E. coli* to classify the strains into pathovars, EPEC (*eaeA*,*lpfA*), animal ETEC (*faeD*), EAEC (*astA* and *pic*), STEC (*stx1*), EHEC (*ehxA*), UPEC (*papC* and *tsh*), and NMEC (*aslA*, *ibeA*, *hek*, *kpsD* and *cdtB*). The majority of the Marmot *E. coli* strains (92.8%) contained multiple pathovar-specific virulence genes. We refer to such strains as pathovar hybrids. The most common pathovar hybrids were a combination of four pathovars, namely EPEC/ETEC/UPEC/NMEC which was detected in 18.4% of the Marmot *E. coli* strains, and EPEC/EAEC/UPEC/NMEC which was detected in 13.6% of the strains (Table 1).

Our whole-genome sequence analysis also revealed that many extensively studied human pathogenic *E. coli* strains also carried virulence genes which were typically belonging to other pathovars (Table 2). The EHEC O157:H7 strains EDL933 and Sakai were found carrying *aslA* gene, frequently detected in NMEC. The UPEC strain CFT073 contained virulence genes such as *astA* and *pic* of EAEC and *aslA* and *kpsD* of NMEC. The EAEC strain 042 contained *lpfA* of EPEC, and *aslA*, *hek* and *kpsD* of NMEC. The ETEC strain H10407 contained *astA* of EAEC and *aslA* of NMEC. The EPEC strain E2348/69 contained *astA* of EAEC and *aslA* of NMEC. The ETEC strain UMNK88 contained *astA* of EAEC and *aslA* of NMEC. The ETEC strain UMNF18 contained *aslA* and *stx2* which are typical NMEC and STEC virulence genes, respectively.

### Phylogenetic relationships of the Marmot *E. coli* virulence genes with those of human pathogenic *E. coli*

**Figure 2:**
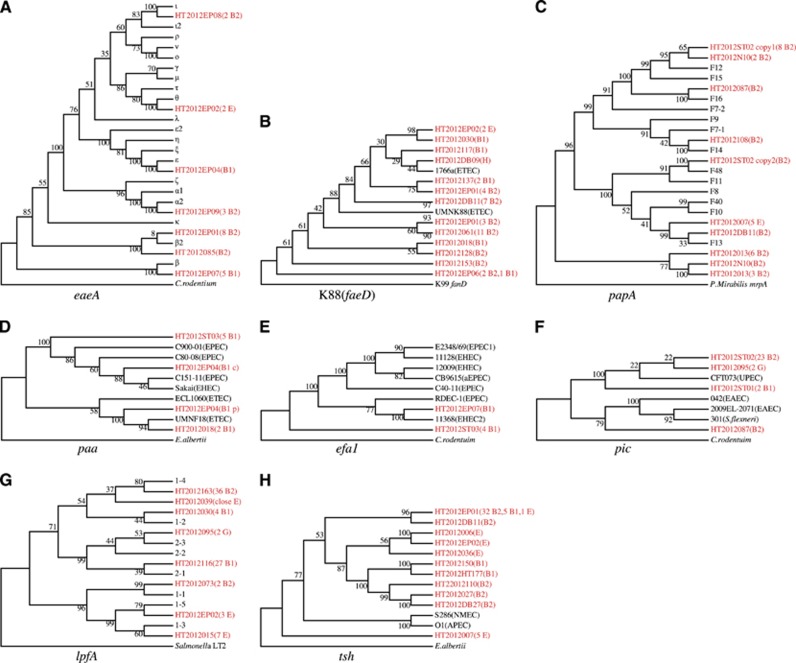
Primordial virulence genes carried by intestinal commensal *E. coli* of *M. himalayana.* (**A–H**) Gene trees for virulence genes with early diverged alleles from Marmot *E. coli*. Each tree represents different virulence genes as indicated. Alleles from Marmot strains were highlighted in red color with strain names followed in brackets by number of strains and the phylogroup of the strain. Alleles on chromosome or plasmid were labeled with c or p, respectively. For *lpfA*, *eaeA* and *papA*, non-Marmot *E. coli* alleles were represented by allele type as classified in published studies. For other genes, a representative strain was used and in brackets following the strain name is pathovar type. The numbers above or below the branches represent percentage bootstrap support.

We extracted DNA sequences of the 22 virulence genes from Marmot *E. coli* genomes and inferred their phylogenetic relationship with human *E. coli* virulence genes. Of 13 genes for which the phylogenetic tree can be rooted with an outgroup, eight were found to contain alleles that were diverged earlier than those from human or other sources, including *eaeA, faeD, papA, paa*, *efa1, tsh, lpfA* and *pic* (Figure 2). Five genes (*aslA*, *ehxA, ibeA*, *hlyA* and *cdtB)* have no earlier diverged alleles observed (Supplementary Figure S1). There was no suitable outgroup for phylogenetic analysis for the remaining nine virulence genes, including *astA, espP, hek, etpD, kpsD, iha, toxB, hlyA* and *shiA* (Supplementary Figure S2).

The *eaeA* gene is located on the LEE pathogenicity island and encodes the intimin adhesin, essential for formation of attaching-and-effacing lesion histopathology of EPEC or EHEC infection.^20^ Six of the 16 known subtypes^21^ were found in Marmot *E. coli*, namely α, β, β2, ɛ, θ and ι. The majority of Marmot *E. coli* strains carried either β (22.3%) or β2 (36.4%) allele. When the sequences from *Citrobacterrodentium* was used as an outgroup, the β-subtype from Marmot *E. coli* HT2012EP07 was the earliest diverged allele (Figure 2A).

The porcine ETEC K88 fimbriae belong to κ-fimbrial clade. The usher gene *faeD* was used to infer the phylogeny. Using K99 fimbriae usher gene *fanD* as an outgroup, the *faeD* allele from Marmot *E. coli* strain HT2012EP06 diverged the earliest. Interestingly, a human ETEC strain 1766a also carried an *faeD,*^22^ which is closely related to the allele from Marmot *E. coli* strain HT2012DB09 (Figure 2B).

P fimbriae are the principal adherence organelles of UPEC. It is antigenically diverse with 11 known serological variants, due to PapA amino acid sequence variation.^23^ When using the *Proteus Mirabilis mrpA* gene encoding the MR/P major fimbrial subunit as an outgroup, a lineage solely composed of Marmot strains diverged the earliest (Figure 2C).

For *paa* encoding the porcine attaching and effacing associated protein, two divergent lineages are known, with one being associated with EPEC and EHEC, while the other with ETEC.^24^ The Marmot *E. coli* HT2012EP04 has two copies of *paa*: one on chromosome and one on a plasmid, which were grouped into the above two lineages. The chromosomal copy from HT2012ST03 was one of the earlier diverged alleles (Figure 2D).

EHEC factor for adherence 1 (encoded by *efa1*) is an essential factor for the adherence to intestinal epithelial cells.^25^ Five Marmot *E. coli* strains carried *efa1*. When the sequence from *C. rodentium* was used as an outgroup, the allele from four Marmot *E. coli* strains diverged earliest (Figure 2E).

The *pic* gene encodes a serine protease and has been reported to be present in UPEC, EAEC and *Shigella.*^26^ When the sequences from *C. rodentium* was used as an outgroup, the alleles from Marmot *E. coli* strains HT2012087 and HT2012ST01 diverged earliest (Figure 2F).

Long polar fimbriae (encoded by *lpfA*) is one of the few adhesive factors of EHEC O157:H7 associated with the colonization of the intestine.^27^ Eight *lpfA* variants exist, namely *lpfA1-1* to *lfpA1-5* and *lpfA2-1* to *lpfA2-3*. When *Salmonella* LT2 was used as an outgroup, *lpfA* from seven Marmot isolates represented by HT2012015 diverged the earliest (Figure 2G).

The hemagglutinin gene *tsh* belong to a subclass of IgA protease family.^28^ When using sequence from *Escherichia albertii* as an outgroup, the *tsh* allele from Marmot *E. coli* strain HT2012007 diverged earlier than that from NMEC and avian pathogenic *E. coli* (APEC) strains (Figure 2H).

### Population structure and evolutionary relationships of Marmot *E. coli* with human and other animal *E. coli*

**Figure 3:**
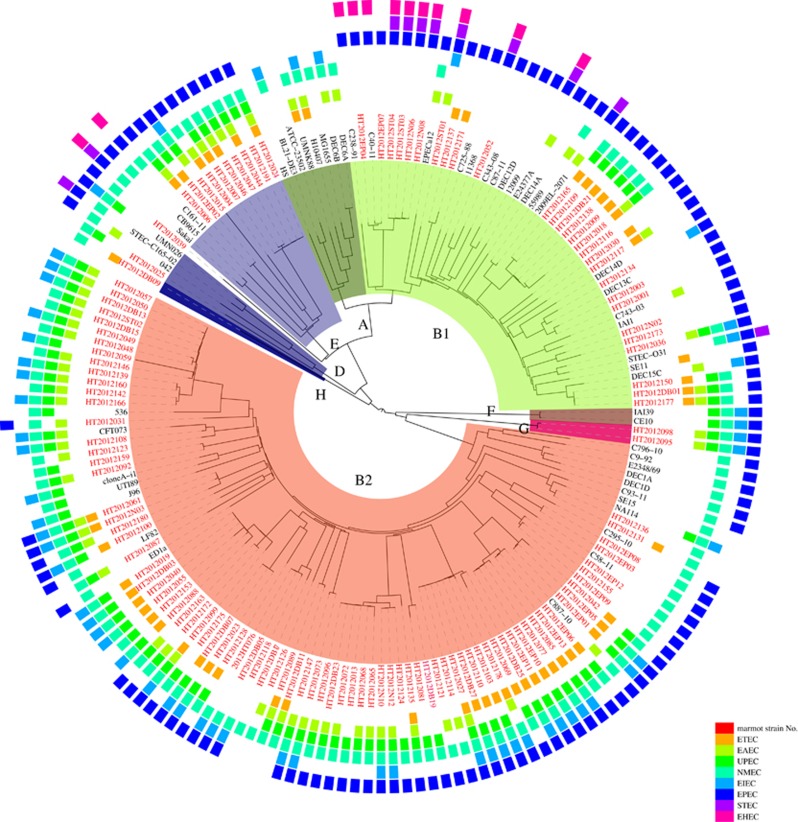
The phylogenetic relationship and virulence gene content of intestinal *E. coli* of *M. himalayana* and 54 representative *E. coli* genomes. The tree was constructed with neighbor-joining algorithm-based 540 core genes. The presence of pathovar-specific genes in each strain was indicated by colored boxes with the color scheme shown. A strain is positive for a specific pathovar if at least one pathovar-specific gene is present. White space represents absence of pathovar-specific genes for a given pathovar in that strain. Strains from Marmots were highlighted in red. Phylogroups were labeled in the inner circle as well as by coloring of the branches.

We analyzed the population structure of *E. coli* with 125 Marmot strains and 355 human and other animal *E. coli* strains which were selected from 1620 genomes available in the GenBank database, with one genome for each sequence type (ST), and one genome for each host of the same ST if multiple genomes were available. Genome comparison found 540 core genes shared by all 480 genomes. Population structure analysis using STRUCTURE^18^ divided the 480 genomes into four groups. Group 1 included phylogroups A and E, each with three subgroups. Group 2 included phylogroups D and F with two subgroups each. Groups 3 and 4 coincided with phylogroup B1 with seven subgroups and B2 with six subgroups respectively. We therefore selected 54 representative strains from these 18 subgroups of four major *E. coli* groups for phylogenetic analysis. Of the 125 Marmot *E. coli* strains, 121 fell into the four known phylogroups with 28 in B1, 82 in B2, 10 in E and one in D. None of the Marmot *E. coli* strains fell into phylogroup A (Figure 3).

Four Marmot *E. coli* strains HT2012DB09, HT2012095, HT2012039 and HT2012098 were not clustered with any known phylogroups. Three of these outlier strains were assigned into two new groups. HT2012095 and HT2012098 were herein assigned to new phylogroup G, while strain HT2012DB09 was assigned to new phylogroup H. Both phylogroups G and H diverged earlier than the other phylogroups. The fourth outlier strain, HT2012039, is distantly related to phylogroup E and is likely to be a new group (Figure 3). The phylogroup G and H strains are distinctive from *Escherichia* clade I and clade V.^29^

**Figure 4:**
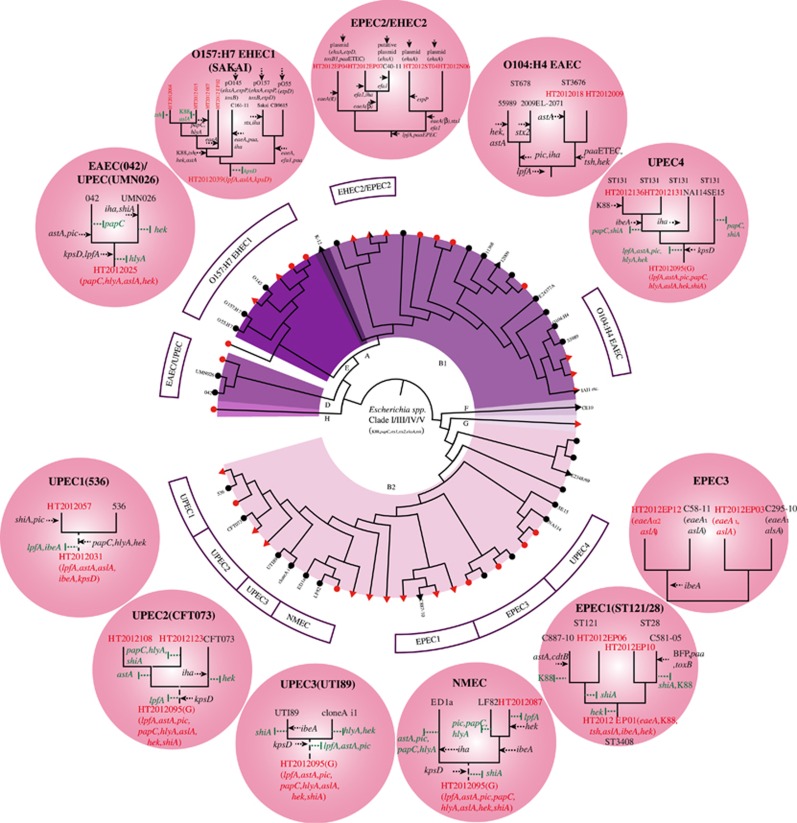
The evolution of major human pathogenic lineages of *E. coli* relative to their closely related Marmot *E. coli* strains. Key non-Marmot *E. coli* strains are given by name and Marmot *E. coli* strains were highlighted with a red dot. Outside the circle in the different shaded circles are a detailed depiction of the pathogenic *E. coli* lineages with gain and loss of genes. Gain of gains is marked by a black arrow, while loss of genes is marked by a vertical green bar. *Escherichia* Clades I–V strains were used as an outgroup.^29^

We further determined the evolutionary relationships of the Marmot *E. coli* strains with major human pathogenic *E. coli* lineages. Phylogenetic analysis of the 125 Marmot *E. coli* strains and 54 representative *E. coli* strains from other sources revealed that 12 major lineages of human pathogenic *E. coli* contained Marmot *E. coli* strains, namely EHEC1, EHEC2, UPEC1-UPEC3, EPEC1-EPEC3 and NMEC (Figure 4). The lineages EHEC1 and EPEC1 were previously recognized as EHEC O157:H7 and EPEC4 respectively.^10^ In five lineages, there were Marmot *E. coli* strains that were diverged earlier than human *E. coli* strains. When the virulence genes within each lineage were compared, we found four patterns of evolution into human pathogens (Figure 4). However, nearly in all cases, there is also gain of virulence genes that are not typical for a given pathogenic type. Some virulence genes were variably present in a lineage and it is difficult to determine whether they were lost in the human strains or gained by other *E. coli* strains within the lineage.

The first pattern is an ancestral multi-pathovar hybrid evolving into a new specific human pathovar by gaining new pathovar-specific genes while retaining other pathovar genes. The O157:H7 lineage shared the most recent common ancestor with Marmot *E. coli* strain HT2012039. The ancestor can be inferred to be an EPEC/NMEC hybrid which acquired EHEC pathogenic specific genes (*stx1/stx2, ehxA*) to gain a new type of pathogenicity. However it still retained *EPEC* and *NMEC* genes. Similarly EPEC1 has an ancestral background of EPEC/UPEC/NMEC and one of the human strains (C581-05) gained additional EPEC virulence genes. However the other human strain (C887-10) has gained no additional EPEC-relevant virulence genes, which fits into pattern 4 below.

The second pattern is multi-pathovar hybrids evolving into a specific pathovar with gain of new pathovar-specific genes and at the same time a reduction of other pathovar genes. UPEC1 has evolved from an EPEC/EAEC/NMEC background but lost *EAEC* genes.

The third pattern is an ancestral single pathovar evolving into the same or different pathovar with gain of pathovar-specific genes. The ancestor of UPEC3 is an NMEC, based on the common virulence properties of the human UPEC strains NA114 and SE15 with Marmot *E. coli* strains. The gain of UPEC specific genes (for example, *papC*) by the human strains led to UPEC pathogenicity. The human strains retained NMEC genes and became a pathovar hybrid. The EPEC1 lineage showed a similar pattern. The ancestor carried common EPEC/UPEC/NMEC genes and one of the human EPEC strain gained EPEC specific *paa* gene during specialization.

The fourth pattern is that the human pathogen shares the same virulence gene profile with Marmot *E. coli,* suggesting a direct spread from wild animals to humans. The EPEC2/EHEC2 lineage contained four Marmot *E. coli* strains, all of which belonged to ST342. Three of the four Marmot *E. coli* strains carried the *ehxA* gene on a plasmid which is shared by the human AEEC (attaching and effacing *E. coli*) strain C40-11. Similarly in the EPEC3 lineage, human EPEC strains share the same EPEC/NMEC virulence gene profile as the Marmot *E. coli* strains. The ancestor of UPEC2 was an EAEC/UPEC/NMEC hybrid and the human UPEC strains retained the same hybrid virulence profile. Similarly the NMEC lineage has an ancestral NMEC background and remained the same when becoming a human pathogen. Some of these lineages (EPEC2/EHEC2, UPEC3) may have been a direct spread from wild animals to humans as they belong to the same STs (for example, the EPEC2/EHEC2 and UPEC3 lineages) and contain the same virulence gene profiles.

## DISCUSSION

Domestic and wild animals have been shown to be the reservoir of human pathogenic *E. coli*. All except EIEC and *Shigella* have been found in other animals where they normally do not cause disease. There has been extensive sampling from domestic animals for pathogenic *E. coli.*^30^ However, sampling from wild animals that have been separated from human activities has been very limited.^31, 32, 33^ In this study we sampled *E. coli* from *M. himalayana* from the Qinghai–Tibet plateau of China which is a relatively pristine environment with little human interference. The Himalayan region is in the high-frigid and meadow-steppe with elevations of 2700–5450 meters and sparse vegetation. The area is sparsely populated with humans, although human settlement traces back to thousands of years ago. The high altitude and harsh climate in the winter make it less suitable for human inhabitation. There are many wild animal species in the region and the species of interest in this study is *M. himalayana.* Of the 14 *Marmota* species, only *M. himalayana* is found in the Himalayan region. *M. himalayana* is the major animal reservoir of *Yersinia pestis* in China and the Qinghai–Tibet plateau plague focus is still active with human infections and deaths in recent years.^34^ For more than half a century, hunting or trading of *M. himalayana* has been banned under the national policy for the control of plague,^35^ which has helped maintain the pristine environment. The *E. coli* we sampled was more likely to be the native population colonizing *M. himalayana*, rather than ingression from humans or domesticated animal species. We showed that all intestinal *E. coli* strains of *M. himalayana* carried virulence genes and are potentially pathogenic to humans. Importantly, the Marmot *E. coli* carried an ancient virulence gene pool as alleles of eight key virulence genes from these *E. coli* strains diverged earlier than alleles of *E. coli* from other sources.

The Marmot *E. coli* strains were not a recent ingression from humans or domestic animal hosts based on the antibiotic resistance profiles. There is a higher prevalence of antibiotic resistance in human *E. coli* population than that of wild animals.^30^ Our Marmot *E. coli* strains were sensitive to all 23 antibiotics tested (data not shown), including penicillins (ampicillin and piperacillin), β-lactam/β-lactamase inhibitor combinations (amoxicillin-clavulanic acid and ampicillin-sulbactam), cephalosporins (cefepime, cefotaxime, ceftriaxone, cephalothin and cefuroxime), monobactams (aztreonam), carbapenems (imipenem and meropenem), aminoglycosides (gentamicin, kanamycin and streptomycin), tetracycline, fluoroquinolones (ciprofloxacin, norfloxacin and levofloxacin), nalidixic acid, trimethoprim-sulfamethoxazole, chloramphenicol and nitrofurantoin. Therefore, the Marmot *E. coli* we sampled was most likely the natural native population of the intestinal flora of the Marmot. The chance of contamination by human *E. coli* was also low as human population density is low at 1.43 persons/km^2^. The distance from sampling sites to nearest human residence village is 10 km to 15 km.

In sharp contrast to what have been observed in human pathogenic *E. coli* that most strains carried pathovar-specific virulence genes,^1, 10, 36^ the Marmot *E. coli* strains have been found to be mostly hybrid forms of carrying virulence genes from different pathovars. It has been considered to be the exception rather than the rule for hybrid forms of *E. coli* with the emergence of STEC/EAEC O104:H4 that caused the German outbreak,^6^ Other EHEC/EAEC hybrid strains, STEC/ETEC, UPEC/STEC, and UPEC/EAEC forms have also been reported recently.^7, 8, 9^ These reports suggest that such hybrid forms exist as human pathogens but are relatively infrequent. In comparison, most of the Marmot *E. coli* strains are hybrids of two or more forms. Although assigning a strain to a pathovar based on one or a few genes is very simplistic and the hybrid forms may not cause clinical disease in humans in multiple forms, our results showed a complex picture of virulence in *E. coli* and high fluidity of virulence genes in the Marmot *E. coli* population. It will be interesting to examine *E. coli* from other wild animals to see whether this is a general picture. Our data also indicated that during their evolution to become human pathogens, modern pathogenic *E. coli* may have lost virulence genes not needed for the specific type of pathogenicity. However it should also be noted that humans may be an accidental host for the various forms of pathogenic *E. coli.*^37, 38^ These genes may have a very different role for the *E. coli* living in its other hosts such as Marmots or the environment.

Our data suggest that some modern human pathogenic *E. coli* may have been derived from ancient pathogens by specialization rather than acquisition of a full set of virulence genes by commensal non-pathogenic *E. coli* to become single mode pathogens. Many pathogens such as O157:H7 have an ancestral pathogenic background with their essential virulence genes already present in their ancient progenitor. Our findings are supported by earlier studies that suggest virulence gene acquisition and expression by human pathogenic *E. coli* require certain phylogenetic background^39^ and that extraintestinal virulence is an ancestral character within the B2 phylogenetic group.^37^ Our results crystalize the previous findings and revealed different patterns of derivation of modern human pathogens from the primordial pathogens.

The distribution of *E. coli* phylogroups has been extensively studied with differential prevalence of the phylogroups in different hosts or human population of different regions.^30^ The Marmot *E. coli* strains predominantly belonged to B2 group with 67.8% and then B1 group with 23.1%. Studies of Australian native animal also showed wild animals harbor predominantly B2 strains with over 30%, while domestic animals harbor predominately B1 strains.^30^ *E. coli* phylogroups D, E, B1 and B2 diverged about 10 million years ago and underwent major expansion about five million years ago.^40^ This timeline is within the time frame when Marmots diverged from squirrels about six million years ago,^41, 42^ suggesting that *E. coli* had evolved into these major phylogroups before they colonized Marmots. In addition, we identified two new phylogroups G and H with the latter being the earliest diverged phylogroup.

This study found that none of Marmot *E. coli* strains fell into phylogroup A. In contrast, phylogroup A is present in high frequency in *E. coli* from human fecal samples, varying from 20% to 77% depending on the geographical region.^30^ Phylogroup A is known to have diverged the latest among the phylogroups.^40, 43^ By virulence gene content, phylogroup A contained the least number of virulence factors.^12^ Indeed the well-known commensal *E. coli* strains in phylogroup A, HS, K-12 and BL21, contained none, one (*aslA*) and three (*aslA, kpsD* and *shiA*) of the 22 virulence genes, respectively. It seems that phylogroup A has undergone virulence gene shedding. Therefore, it is likely that human commensal *E. coli* may be an ultimately evolved rather than the primitive form of *E. coli*.

In conclusion, Marmot *E. coli* carried many virulence genes in a mixed virulence gene pool and hybrid pathogenic forms were found which have the potential to cause disease in humans. Two new phylogroups, H and G, both of which diverged earlier than the other phylogroups were identified. The Marmot *E. coli* population seemed to carry an ancient virulence gene pool. Eight of the 12 well-known pathogenic *E. coli* lineages shared a most recent common ancestor with one or more Marmot *E. coli* strains. These findings provided a new understanding of the evolutionary origin of pathogenic *E. coli*.

## Figures and Tables

**Table 1:** Pathovar hybrid patterns of 125 Marmot *Escherichia coli* strains

**Pathovar hybrid pattern**	**Number of strains**	**Percentage of strains**
EPEC/ETEC/UPEC/NMEC/EIEC	13	10.40
EPEC/ETEC/UPEC/NMEC	10	8.00
EPEC/ETEC/EAEC/UPEC/NMEC	9	7.20
EPEC/EAEC/UPEC/NMEC	9	7.20
EPEC/EAEC/UPEC/NMEC/EIEC	8	6.40
EAEC/UPEC/NMEC/EIEC	8	6.40
EPEC/UPEC/NMEC	8	6.40
EAEC/UPEC/NMEC	7	5.60
EPEC/NMEC	6	4.80
ETEC/UPEC/NMEC/EIEC	5	4.00
EPEC/ETEC/EAEC/UPEC/NMEC/EIEC	5	4.00
EPEC/STEC/EHEC	4	3.20
EPEC/EAEC/NMEC	3	2.40
EPEC/NMEC/EIEC	3	2.40
EPEC/ETEC	3	2.40
EAEC/NMEC	2	1.60
ETEC/EAEC/UPEC/NMEC	2	1.60
ETEC/NMEC	2	1.60
UPEC/NMEC	2	1.60
EPEC/EHEC	1	0.80
EPEC/EIEC	1	0.80
EPEC/ETEC/EAEC	1	0.80
EPEC/ETEC/EAEC/NMEC	1	0.80
EPEC/STEC/EHEC/EAEC/EIEC	1	0.80
UPEC/NMEC/EIEC	1	0.80
ETEC/UPEC/NMEC	1	0.80
EPEC	7	5.60
NMEC	2	1.60
Total	125	100

**Table 2:** Presence of virulence genes of different pathovars in well-known typical pathogenic *E.coli* strains

*Pathovar type*	*Strain*	*Virulence genes*^a^																					
		*EPEC*				*EHEC*						*ETEC*	*EAEC*		*UPEC*			*NMEC*					*EIEC*
		*eaeA*	*lpfA*	*efa1*	*paa*	*stx*	*ehxA*	*espP*	*etpD*	*toxB*	*iha*	*K88*	*astA*	*pic*	*papC*	*hlyA*	*tsh*	*aslA*	*ibeA*	*hek*	*kpsD*	*cdt*	*shiA*
EHEC	EDL933	1	1	0	1	1	1	1	1	1	1	0	0	0	0	0	0	1	0	0	0	0	0
EHEC	Sakai	1	1	0	1	1	1	1	1	1	1	0	0	0	0	0	0	1	0	0	0	0	0
UPEC	CFT073	0	0	0	0	0	0	0	0	0	1	0	1	1	1	1	0	1	0	0	1	0	1
EAEC	42	0	1	0	0	0	0	0	0	0	0	0	1	1	0	0	0	1	0	1	1	0	0
ETEC	H10407	0	0	0	0	0	0	0	0	0	0	0	1	0	0	0	0	1	0	0	0	0	1
EPEC	E2348/69	1	1	1	0	0	0	0	0	0	0	0	1	0	0	0	0	1	0	0	0	0	0
ETEC	UMNK88	0	0	0	0	0	0	0	0	0	1	1	1	0	0	1	0	1	0	0	0	0	0
ETEC	UMNF18	0	0	0	0	1	0	0	0	0	0	0	0	0	0	0	0	1	0	0	0	0	0

a 1: present; 0: absent; for stx: present if either or both stx1 and stx2 are present.
