# Supplementary material for: Insights into the evolution of pathogenicity of Escherichia coli from genomic analysis of intestinal E. coli of Marmota himalayana in Qinghai–Tibet plateau of China
Source: Emerg Microbes Infect. 2016 Dec 7;5(12):e122–. doi: 10.1038/emi.2016.122 (PMC5180367; doi:10.1038/emi.2016.122)
Supplement: Supplementary Figure S2 [file emi2016122x2.pdf]

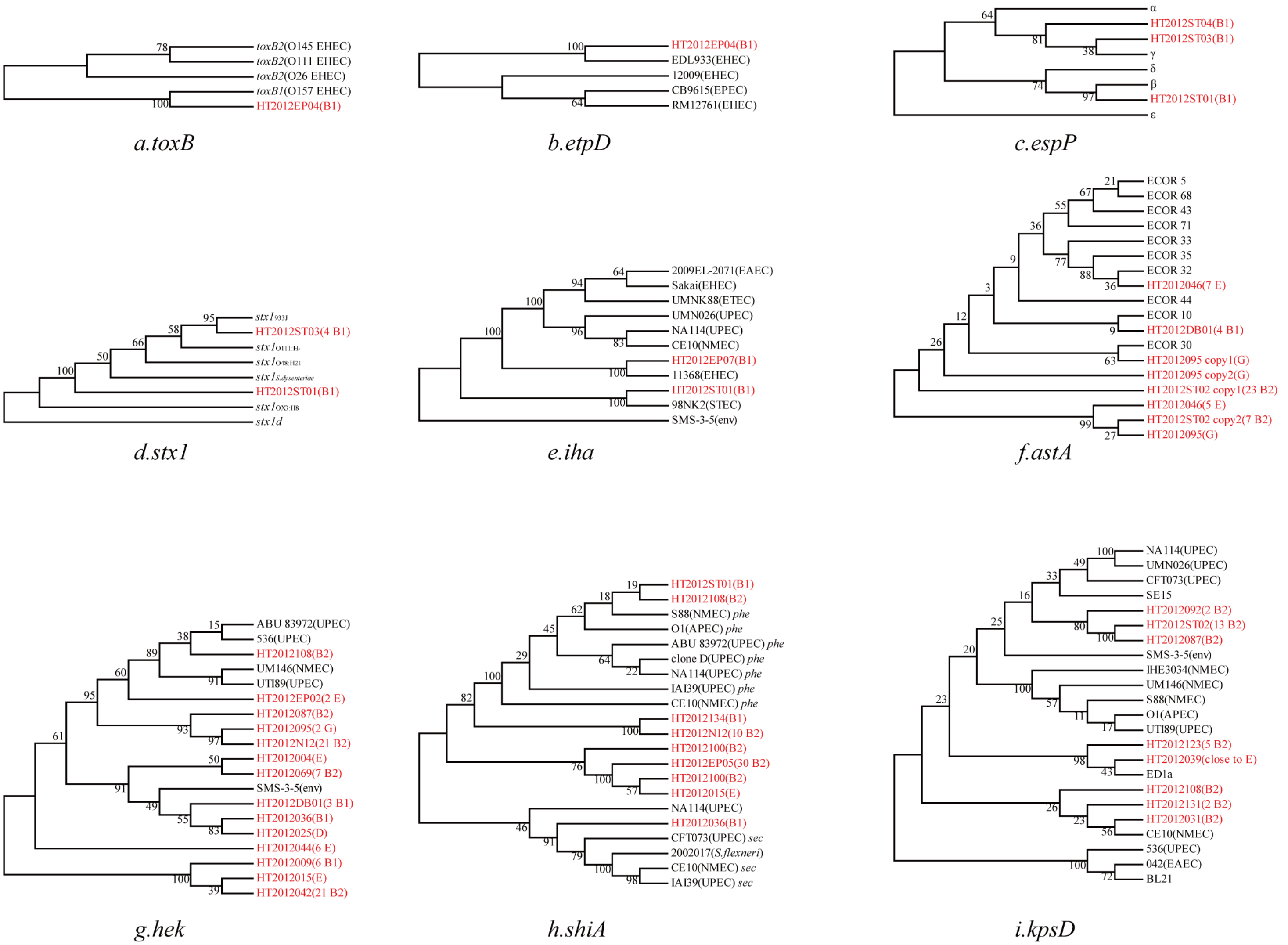

Supplementary Figure S2 The phylogenic analysis of virulence genes without suitable outgroup carried by intestinal *E. coli* of *M. himalayana*. a-i: gene trees for virulence genes with no suitable outgroup. Each tree represents different virulence genes as indicated. Alleles from marmot strains were highlighted in red color with strain names followed in brackets by number of strains and the phylogroup of the strain. Alleles on chromosome or plasmid were labeled with c or p respectively.
